# Narrow-band near-infrared photocurrent enhancement *via* toroidal dipole resonance in Si_1−*x*_Ge_*x*_ nanodisk arrays

**DOI:** 10.1039/d6na00324a

**Published:** 2026-05-08

**Authors:** Nguyen Quoc Chien, Keisuke Moriasa, Hiroshi Sugimoto, Minoru Fujii

**Affiliations:** a Department of Electrical and Electronic Engineering, Graduate School of Engineering, Kobe University Kobe 657-8501 Japan fujii@eedept.kobe-u.ac.jp

## Abstract

Enhancement of light absorption and photocurrent by toroidal dipole resonances in a Si nanodisk array in the near-infrared (NIR) spectral range by controlling both the structural and material parameters has been investigated. To optimize absorption, we introduced Si_1−*x*_Ge_*x*_ alloying (*x* < 0.375) to tune the material loss while maintaining a nearly constant refractive index. Simulations revealed that absorptance does not increase monotonically with Ge content but reaches a maximum at a specific composition, corresponding to the critical coupling condition. Experimental fabrication and characterization confirmed this behavior, showing that a small Ge incorporation (*x* ≈ 0.125) enhances absorptance more than threefold and increases photocurrent up to 3.4 times compared with pure Si metasurfaces. This study demonstrates that alloy engineering provides a practical route to achieve critical coupling and maximize photocurrent in Si-based metasurfaces operating in the NIR region.

## Abstract 2

Si–Ge alloying provides an effective means to control the material loss of the Si-based metasurface and thereby optimizes absorptance at the resonances. Experimental results confirm that small Ge incorporation enhances absorptance and photocurrent.

## Graphical abstract

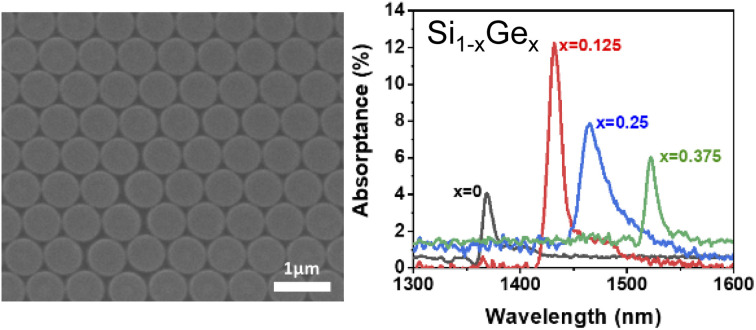

## Introduction

Near-infrared (NIR) light is transparent to many materials and plays a vital role in modern photonic technologies. It has been extensively utilized in optical telecommunications,^1–4^ biomedical imaging,^5–10^ environmental monitoring,^11–13^ and autonomous sensing systems.^14–17^ However, the detection range of conventional silicon (Si) photodetectors is limited to approximately 1100 nm. To extend the operational wavelength beyond the telecom band (∼1550 nm), narrower bandgap semiconductors such as InGaAs,^18,19^ InP,^20^ and Ge^21,22^ have been integrated on Si substrates through wafer bonding or epitaxial growth techniques.

Recently, several strategies have been proposed to extend the detection wavelength range of a Si-based photodetector. One of the promising approaches involves extracting hot electrons generated *via* the damping of localized surface plasmons in gold (Au) nanorods at a Si–Au Schottky barrier.^23–25^ Another strategy is engineering defect states in Si to enhance the sub-bandgap absorption.^26–28^ Enhancing weak defect-related sub-bandgap absorption *via* optical resonances has also attracted considerable attention.^29–34^ For example, narrowband NIR photocurrent enhancement has been achieved in a polycrystalline-Si metasurface composed of a hexagonal array of Si nanodisks.^31,32^ In that work, toroidal dipole (TD) resonances were employed as the resonance mode. Compared with conventional electric dipole (ED) resonances, TD resonances offer stronger confinement of the electromagnetic field and reduced radiation leakage, thereby enabling high-Q resonances.^35–38^

Absorptance enhancement by optical resonances reaches its maximum when the material loss equals the radiation loss, a condition known as critical coupling.^38–40^ In general, the radiation loss rate, *i.e.*, the coupling efficiency of a mode to free-space radiation, in a metasurface can be controlled through its geometric configuration. In contrast, the material loss, determined by the refractive index and the extinction coefficient, is an intrinsic property of the material and is usually difficult to adjust. As a result, most previous studies have focused exclusively on geometric optimization to reduce radiation loss,^41,42^ implicitly assuming fixed material absorption. However, for Si-based materials such as polycrystalline or amorphous Si, the extinction coefficient at telecom wavelengths is very low, making it impractical to achieve critical coupling in simple structures. In such cases, simultaneous control of both radiation loss and material loss provides a practical route for achieving high absorption at optical resonances without increasing the structural complexity.

In this work, to enhance TD resonance-induced absorption in Si nanodisk arrays at around the telecom wavelength, we investigate the effect of the material loss through Si_1−*x*_Ge_*x*_ alloying (*x* < 0.375). Within this composition range, the extinction coefficient varies by more than an order of magnitude, whereas the refractive index changes only slightly. We first employ numerical simulations to examine the influence of structural parameters and alloy composition on the absorption properties. Our results show that the absorptance does not increase monotonously with *x*, but instead reaches a maximum at a specific composition. In the proposed structure, the absorptance increases sharply with increasing *x*, attains its maximum below *x* = 0.1, and then decreases. Therefore, a significant enhancement of absorptance can be achieved by incorporating only a very small amount of Ge into Si. We subsequently fabricate Si_1−*x*_Ge_*x*_ nanodisk arrays with varying Ge contents and investigate their absorption and photocurrent spectra. We demonstrate that a small Ge incorporation increases the photocurrent of the metasurfaces by up to 3.4 times compared with pure Si metasurfaces.

## Results and discussion

**Fig. 1:**
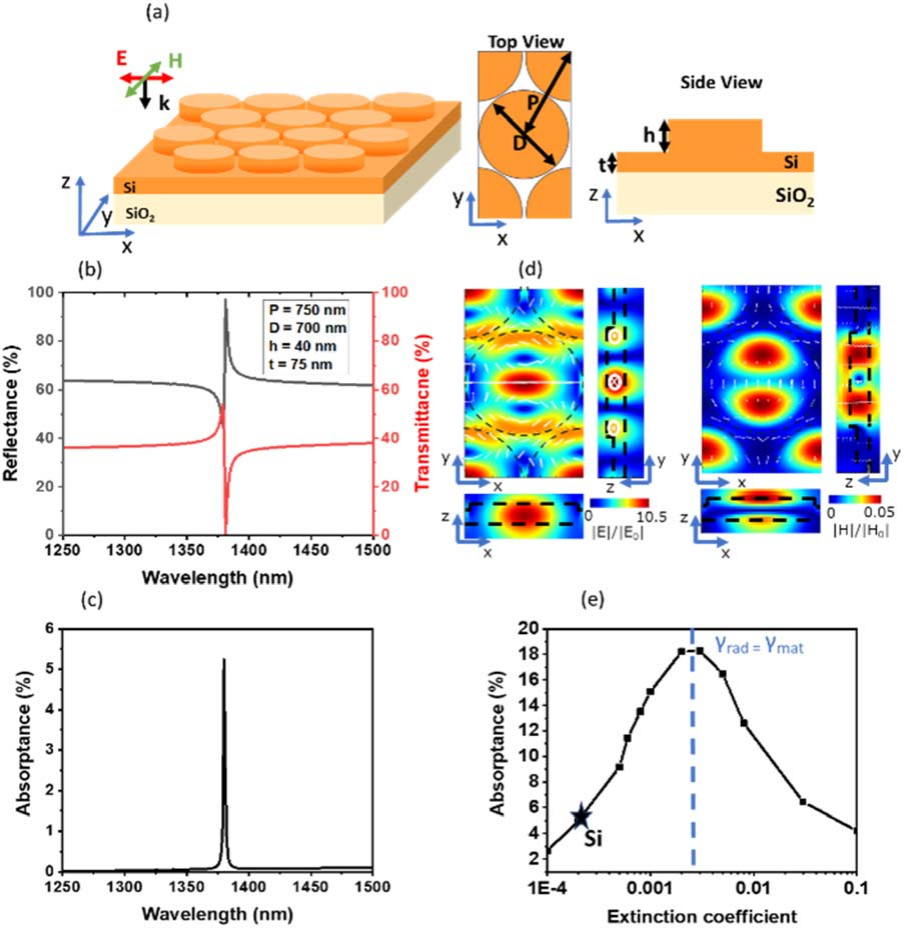
(a) Schematic illustration of a Si nanodisk array. (b) Reflectance (black line), transmittance (red line), and (c) absorptance spectra of a Si nanodisk array with a period (*P*), diameter (*D*), height (*h*) and thickness of the thin film (*t*) of 750 nm, 700 nm, 40 nm, and 75 nm, respectively. (d) Electric and magnetic field distributions of the structure at the resonance peak. (e) Absorptance at the resonance peak as a function of the extinction coefficient (*k*) (10^−4^ – 10^−1^).

Fig. 1(a) shows a schematic illustration of a metasurface composed of a hexagonal array of Si nanodisks formed on a Si thin film. Fig. 1(b) shows calculated transmittance (*T*) and reflectance (*R*) spectra under normal incidence for the structure with a diameter (*D*), periodicity (*P*), and height (*h*) of the nanodisk of 700 nm, 750 nm, and 40 nm, respectively, and a thickness (*t*) of the Si thin film of 75 nm. The refractive index and extinction coefficient used for the calculations are provided in the SI (Fig. S1). The substrate is silica. The absorptance (*A*) obtained from *A* = 1 − *T* – *R* is shown in Fig. 1(c). We can see a sharp resonance at 1380 nm with a *Q*-factor of 650. The electric (|*E*|/|*E*_0_|) and magnetic (|*H*|/|*H*_0_|) field distributions at the resonance wavelength in the *xy*-(*z* = 0), *yz*-(*x* = 0), and *zx*-planes (*y* = 0) are shown in Fig. 1(d). Symmetric current loops and a circulating magnetic field, characteristic of a toroidal dipole resonance, are observed.^31,32,36^ Multipole decomposition of the local fields also indicates a dominant contribution of the TD moment to the resonance (Fig. S2 in the SI). The electric field is tightly confined within Si nanodisks, and the underlying Si thin film, with the electric field enhancement factor reaching 10.5. This strong field confinement enables significant light absorption despite the very small extinction coefficient assumed in the calculation (2 × 10^−4^).^31^

The absorptance at the TD resonance reaches its maximum at the critical coupling condition, where the material loss rate (*γ*_mat_) matches the radiation loss rate (*γ*_rad_).^38–40^ In general, to maximize the absorptance, the radiation loss is tuned through the structural parameters to match the material loss. However, in the sub-bandgap wavelength range of Si, it is not very practical to reduce the radiation loss to achieve the critical coupling condition in a simple structure. Therefore, we focus on controlling the material loss to maximize the absorptance. Fig. 1(e) shows the peak absorptance as a function of the extinction coefficient at a fixed refractive index (*n* = 3.5). The peak absorptance exhibits a clear dependence on the extinction coefficient, and for the present structural parameters, it reaches a maximum at *k* = 0.003, indicating the satisfaction of the critical coupling condition. Structures with different geometrical parameters show a similar dependence, as presented in the SI (Fig. S3(a)). In contrast, the *Q*-factor decreases monotonically with increasing *k* (SI (Fig. S3(b))).

**Fig. 2:**
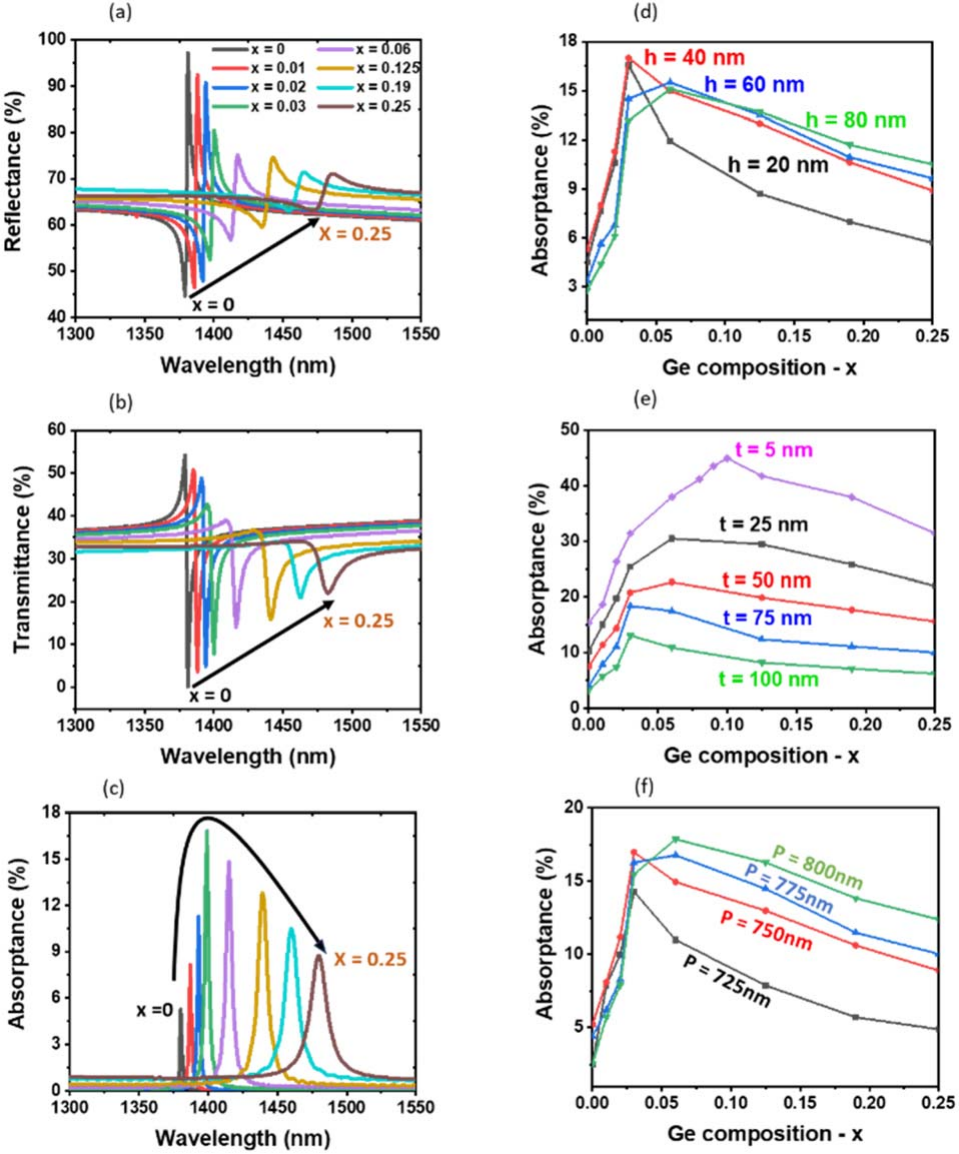
(a) Reflectance, (b) transmittance, and (c) absorptance spectra of Si_1−*x*_Ge_*x*_ nanodisk arrays with *x* ranging from 0 to 0.25, for *h* = 40 nm, *P* = 750 nm, *D* = 700 nm, and *t* = 75 nm. (d–f) Peak absorptance as a function of Ge composition: (d) *h* varies from 20 nm to 80 nm, with *P* = 750 nm, *D* = 700 nm, and *t* = 75 nm. (e) *t* varies from 5 nm to 100 nm, with *h* = 40 nm, *P* = 750 nm, and *D* = 700 nm. (f) *P* varies from 725 nm to 800 nm, with *h* = 40 nm, *D* = 700 nm and *t* = 75 nm.

To control the extinction coefficient without significantly affecting the refractive index, we employ a Si_1−*x*_Ge_*x*_ alloy with varying *x*. The refractive index and the extinction coefficient of the Si_1−*x*_Ge_*x*_ alloy (0 < *x* < 0.25) used for simulations are shown in the SI (Fig. S1). Within this composition range, the extinction coefficient increases sharply; for example, at a wavelength of 1500 nm, *k* increases from 1.2 × 10^−4^ to 7 × 10^−3^ – an almost 60-fold increase with increasing *x*, whereas the refractive index increases by only a factor of 1.1 The extinction coefficient of the Si_1−*x*_Ge_*x*_ alloy in this range is suitable to achieve the critical coupling condition of TD resonance (Fig. 1(e)). We calculated the reflectance and the transmittance as a function of *x* for different structural parameters. Fig. 2(a–c) shows calculated reflectance, transmittance, and absorptance spectra, respectively, for the structure with *h* = 40 nm, *P* = 750 nm, *D* = 700 nm, and *t* = 75 nm. As *x* increases from 0 to 0.25, the TD resonance redshifts due to the slight increase in the refractive index. Simultaneously, the resonance becomes broader and weaker owing to the rapid increase in the extinction coefficient and the resultant increase in the material loss rate.

In contrast to the monotonous changes in reflectance and transmittance with *x*, the absorptance first increases, reaches a maximum at around *x* = 0.03, and then decreases. A similar *x* dependence of the absorptance is observed for different structural parameters. Fig. 2(d–f) shows the *x* dependence of absorptance for structures with varying *h*, *t*, and *P*, respectively. In all the structures, absorptance reaches its maximum in the range *x* = 0.03–0.10. The slight differences in the optimum *x* among the structures can be attributed to differences in the radiation loss rate. For example, as shown in Fig. 2(d), increasing the disk height, *h*, enhances the radiation leakage, thereby requiring a higher material loss to satisfy the critical coupling condition; this shifts the optimum *x* toward larger values. On the other hand, as *t* increases (Fig. 2(e)), the optimum *x* shifts toward smaller values due to stronger field confinement within a film and resulting reduction in radiation leakage. The optimum *x* also depends on *P* (Fig. 2(f)), where increasing *P* reduces inter-nanodisk coupling. This leads to a decrease in the resonance *Q*-factor, shifting the optimum *x* toward larger values. In the SI (Fig. S4), we also show D-dependence of the peak absorptance under the condition of *P* – *D* = 50 nm. While *D* is the dominant factor in determining the resonance wavelength, it does not strongly affect the optimum *x*. Overall, tuning the material loss *via* the Ge content and the radiation loss *via* structural parameters provides a means to approach the critical coupling condition for the TD resonance at the telecom band.

Notably, the *Q*-factor of the resonance decreases monotonously with increasing *x* (see Fig. S5 in the SI). This behaviour arises because the *Q*-factor is determined by the sum of the material loss rate (*γ*_mat_) and the radiation loss rate (*γ*_rad_). These results demonstrate that maximum absorptance stems from balancing material and radiation losses, rather than simply increasing the material loss.

**Fig. 3:**
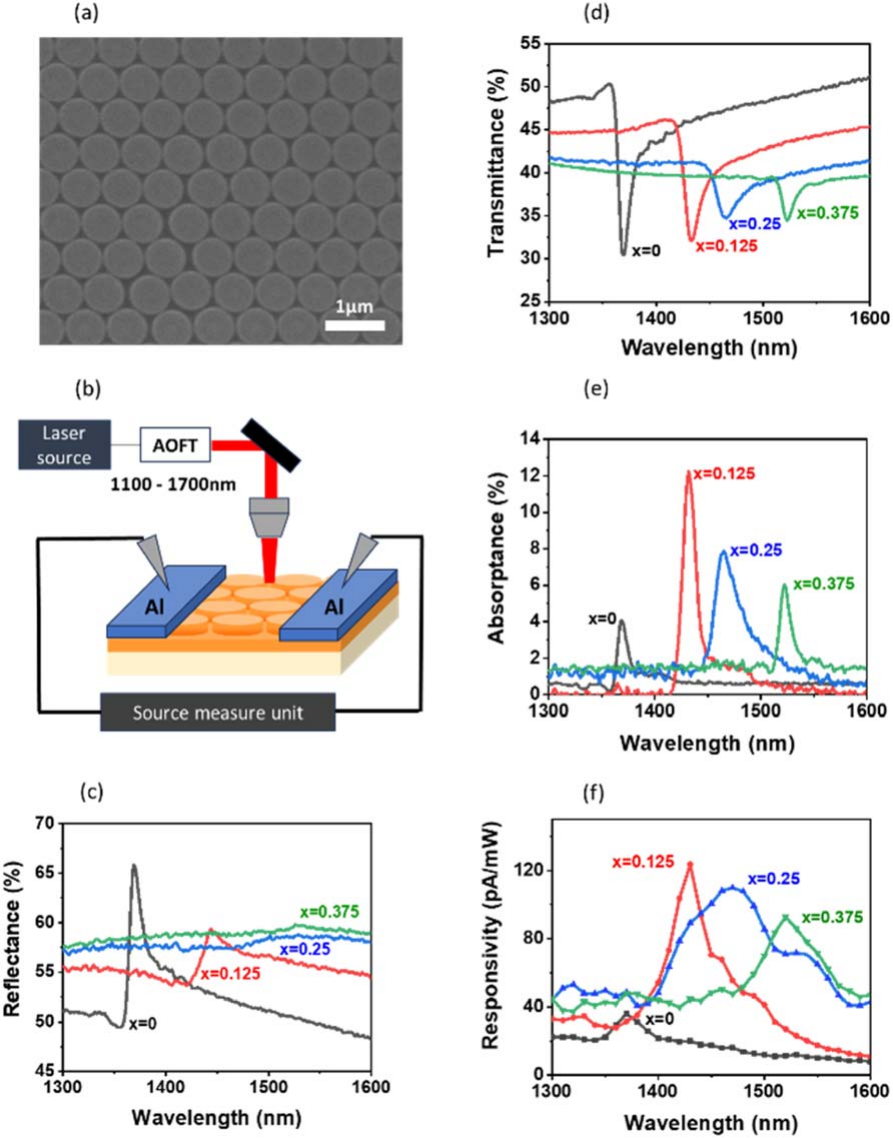
(a) SEM image of the Si_0.875_Ge_0.125_ nanodisk array. (b) Schematic illustration of the photocurrent measurement setup. (c) Reflectance, (d) transmittance, (e) absorptance, and (f) photo-responsivity spectra of Si_1−*x*_Ge_*x*_ nanodisk arrays with *x* ranging from 0 to 0.375 for *P* = 750 nm, *D* = 700 nm, *h* = 40 nm, and *t* = 75 nm.

Following the simulations, we fabricated Si_1−*x*_Ge_*x*_ nanodisk arrays on silica substrates. The structural parameters are *P* = 710 nm, *D* = 550 nm, *h* = 30 nm, and *t* = 75 nm, and the Ge composition is changed from 0 to 0.375. Fig. 3(a) shows the scanning electron microscope (SEM) image of a structure with *x* = 0.125. The atomic force microscope (AFM) image is provided in the SI (Fig. S7). On a nanodisk array, aluminium (Al) stripe electrodes are formed for the photocurrent measurements (Fig. 3(b)). Fig. 3(c) and (d) show the measured reflectance and transmittance spectra. With increasing *x*, the TD resonance shifts to a longer wavelength and becomes weaker, in agreement with the simulations. A comparison between the measured and calculated spectra is shown in the SI (Fig. S8). The overall spectral shape agrees well, although the measured spectra are slightly broader than the calculated ones.

Fig. 3(e) presents the absorptance spectra obtained from the reflectance and transmittance spectra. A non-monotonic dependence of the absorptance on *x* is clearly observed. The absorptance increases more than threefold at *x* = 0.125 compared with a Si nanodisk array with the same structural parameters. It then decreases with further increasing *x*, reaching half of the maximum at *x* = 0.375 despite the increases in the extinction coefficient. This suggests that the critical coupling condition is satisfied around the absorptance maximum.

Finally, we measured the photo-responsivity spectra of the same nanodisk arrays. The results are shown in Fig. 3(f). We can see resonance peaks around the absorptance maxima, indicating that photocurrent is enhanced *via* the TD resonance of Si_1−*x*_Ge_*x*_ alloy nanodisks. At *x* = 0.125, the photocurrent is enhanced by a factor of 3.4 compared with a pure Si nanodisk array. It is important to note that the observed photocurrent enhancement is not simply due to increased intrinsic absorption of Si_1−*x*_Ge_*x*_ alloy films (see Fig. S9 in the SI), but predominantly originates from resonance-enhanced absorption associated with the improved critical coupling condition achieved through Ge-induced tuning of the complex permittivity. In fact, the enhancement factor of the peak photocurrent with respect to the background reaches the maximum at *x* = 0.125, and then decreases with further increases in *x*. This trend closely follows that of the absorptance. Therefore, controlling the extinction coefficient of a material to achieve a critical coupling condition is an effective strategy for maximizing photocurrent in a metasurface.

In this work, the enhancement factor of the photocurrent with respect to the background signal is relatively small compared to that of the absorptance. This may largely be due to the non-optimized device structure. The large electrode spacing (∼100 µm) requires a long transport distance for photogenerated carriers. Given the very low aspect ratio of the metasurfaces, carrier recombination and scattering at the surfaces play a significant role. These effects limit the collection efficiency, especially when carrier generation is highly localized at the TD resonances, preventing the full translation of absorption enhancement into photocurrent. The relatively large bandwidth (ranging from 6.4 to 19.8 nm) of the excitation light for photocurrent measurements also causes the broadening of and decrease in the resonance peak.

## Conclusions

We have demonstrated that Si_1−*x*_Ge_*x*_ alloying provides an effective means to control the material loss of the Si-based metasurface and thereby optimize absorptance at TD resonances around the telecom wavelength. Numerical simulations revealed that absorptance does not increase monotonically with Ge content but instead reaches a maximum at a specific composition, corresponding to the critical coupling condition. Experimental fabrication and characterization of Si_1−*x*_Ge_*x*_ nanodisk arrays confirmed this prediction, showing that incorporation of a small amount of Ge (*x* ≈ 0.125) enhances absorptance and photocurrent by more than threefold compared with the pure Si metasurface.

These results highlight the importance of simultaneous control of radiation loss and material loss to achieve high-Q resonances and efficient light absorption in sub-bandgap Si-based photonic structures. The demonstrated strategy of tuning the extinction coefficient through alloy engineering offers a practical pathway for enhancing the performance of Si-compatible photodetectors and metasurfaces operating in the near-infrared region. Beyond photodetection, this approach may be extended to other optoelectronic applications, including energy harvesting, nonlinear optics, and on-chip sensing, where precise control of optical resonances and absorption is essential.

## Experimental procedure

Si_1−*x*_Ge_*x*_ nanodisk arrays were fabricated by nanosphere lithography (see the SI (Fig. S6)). First, Si_1−*x*_Ge_*x*_ films with thicknesses ranging from 70 to 130 nm were deposited onto a 750 µm-thick SiO_2_ substrate (2 cm × 2 cm) using an RF sputtering system (ANELVA: SPF-210H). The Ge concentration was determined from the Raman scattering spectra (see the SI (Fig. S10)). Subsequently, a monolayer of 750 nm-diameter polystyrene beads (PSBs; Polysciences Inc.) was formed on the alloy surface as an etching mask. The PSB diameter, which defines the mask size, was reduced to targeted values by oxygen plasma (ANELVA: L-201D). The mask pattern was then transferred onto Si_1−*x*_Ge_*x*_ films *via* Ar^+^ etching. The etching time was controlled to obtain a desired disk height. After removing the PSB mask using *N*,*N*-dimethylformamide (FUJIFILM Wako), the samples were annealed at 800 °C in an N_2_ atmosphere for 2 hours to induce crystallization. For the photocurrent measurements, aluminium electrodes (400 µm × 200 µm, thickness 100 nm, spacing 100 µm) were fabricated by vacuum deposition after removing the surface native oxide by HF etching. The samples were then sintered at 600 °C for 10 min in a N_2_ atmosphere.

Reflectance and transmittance spectra of the nanodisk arrays were recorded using a double-beam spectrophotometer (SHIMADZU, UV-3101PC) with randomly polarized incident light. For photocurrent measurements, a nanodisk array was illuminated from the normal to the surface through an objective lens (N.A. = 0.055). The illumination source was a supercontinuum laser (NKT Photonics, SuperK EVO, beam size ∼2 mm) spectrally filtered by an acousto-optic tuneable filter (SUPERK SELECT IR, 1100–1700 nm, bandwidth 6.4–19.8 nm). The photocurrent was measured using a source measurement unit (Keithley 236) under an applied bias of 100 V. All photocurrent measurements were conducted in a vacuum.

## Author contributions

The manuscript was written through the contributions of all authors. All authors have given approval to the final version of the manuscript.

## Conflicts of interest

There are no conflicts to declare.

## Supplementary Material

NA-008-D6NA00324A-s001

## Data Availability

The data supporting this article are available from the corresponding author upon reasonable request. Supplementary information (SI) is available. See DOI: https://doi.org/10.1039/d6na00324a.
