# Supplementary material for: Narrow-band near-infrared photocurrent enhancement via toroidal dipole resonance in Si1−xGex nanodisk arrays
Source: Nanoscale Adv. 2026 May 8;8(12):3581–6. doi: 10.1039/d6na00324a (PMC13207905; doi:10.1039/d6na00324a)
Supplement: NA-008-D6NA00324A-s001 [file NA-008-D6NA00324A-s001.pdf]

Supplementary Information (SI) for Nanoscale Advances.
This journal is © The Royal Society of Chemistry 2026

Supporting information

Narrow-Band Near-Infrared Photocurrent Enhancement via Toroidal Dipole Resonance in $Si_{1-x}Ge_x$ Nanodisk Array

Nguyen Quoc Chien, Keisuke Moriasa, Hiroshi Sugimoto, and Minoru Fujii.

Department of Electrical and Electronic Engineering, Graduate School of Engineering, Kobe University, Kobe 657-8501, Japan
E-mail: sugimoto@eedept.kobe-u.ac.jp
fujii@eedept.kobe-u.ac.jp

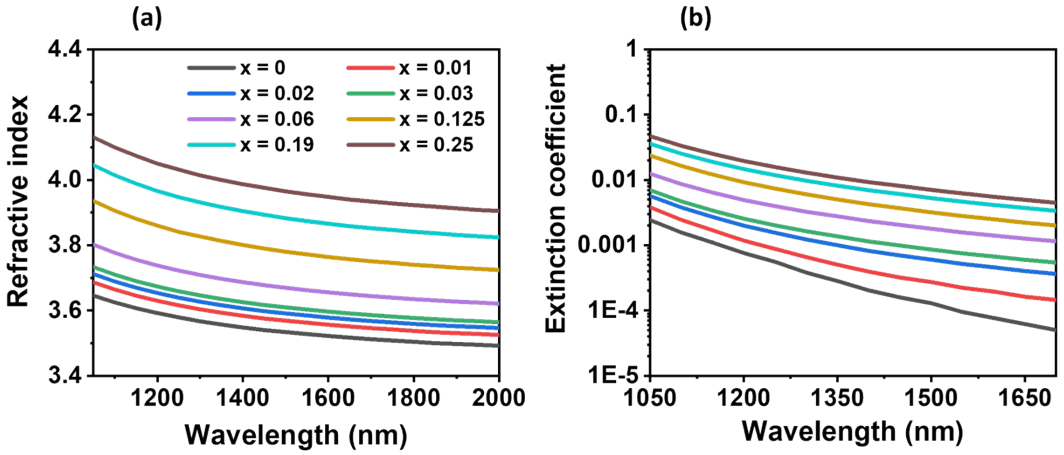

Figure S1. (a) Refractive index and (b) extinction coefficient of $Si_{1-x}Ge_x$ alloy with x ranging from 0 to 0.375. The data are obtained from DFT-based SIESTA calculations using large grain-boundary supercells, which represent the polycrystalline structure by incorporating atomic disorder and local bonding variations at the grain interfaces.

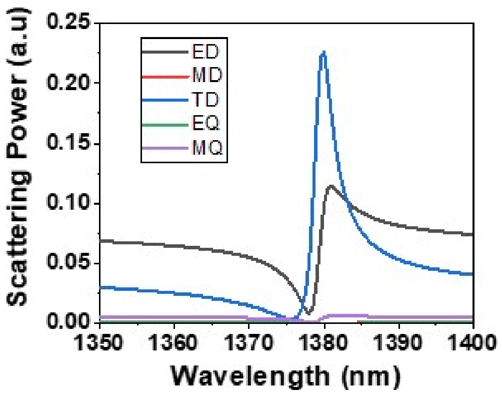

Figure S2. Scattering power spectra of Cartesian ED, MD, MQ, and TD moments obtained by multipole decomposition of the induced current in the nanodisk array in Figure 1.

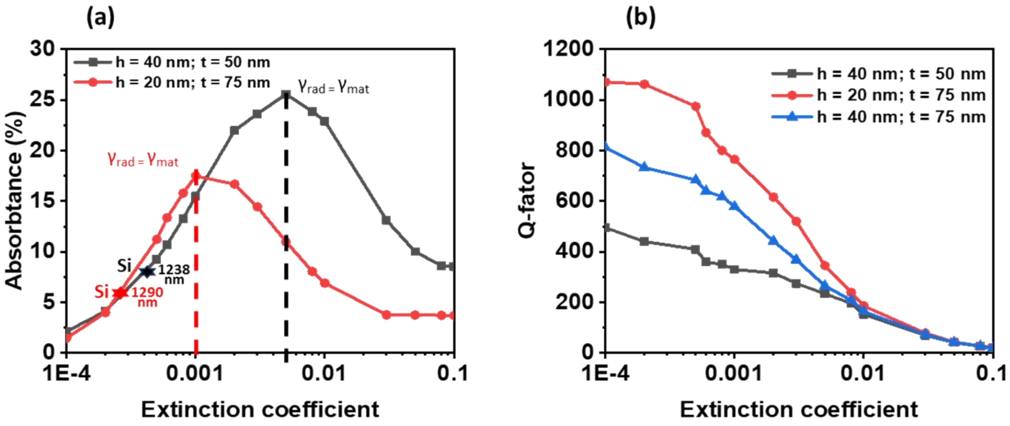

Figure S3. (a) Absorptance at the resonance peak as a function of extinction coefficient for two geometries: h = 40 nm, t = 50 nm (black), and h = 20 nm, t = 75 nm (red), with n = 3.5, P =750 nm, and D = 700nm. (b) Q-factor of the peak as a function of extinction coefficient for three geometries: when h = 40 nm, t = 50 nm (black), h = 20 nm, t = 75 nm (red), and h = 40 nm, t = 75 nm (blue), with n = 3.5, P =750 nm and D = 700nm.

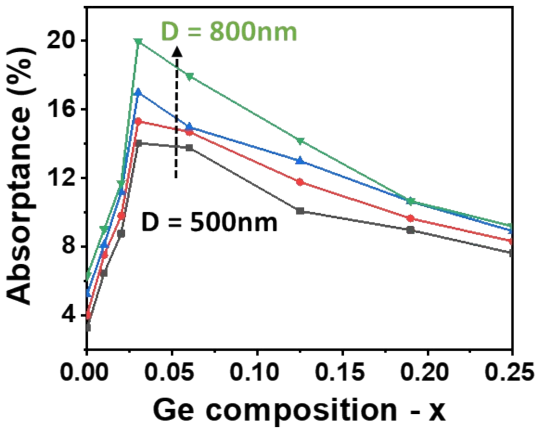

Figure S4. Absorptance at the resonance peak of $Si_{1-x}Ge_x$ nanodisk arrays for composition x ranging from 0 to 0.25, when the disk diameter (D) varies from 500 nm to 800 nm. Other parameters are fixed at h = 40 nm, t = 50 nm, and P = D + 50 nm.

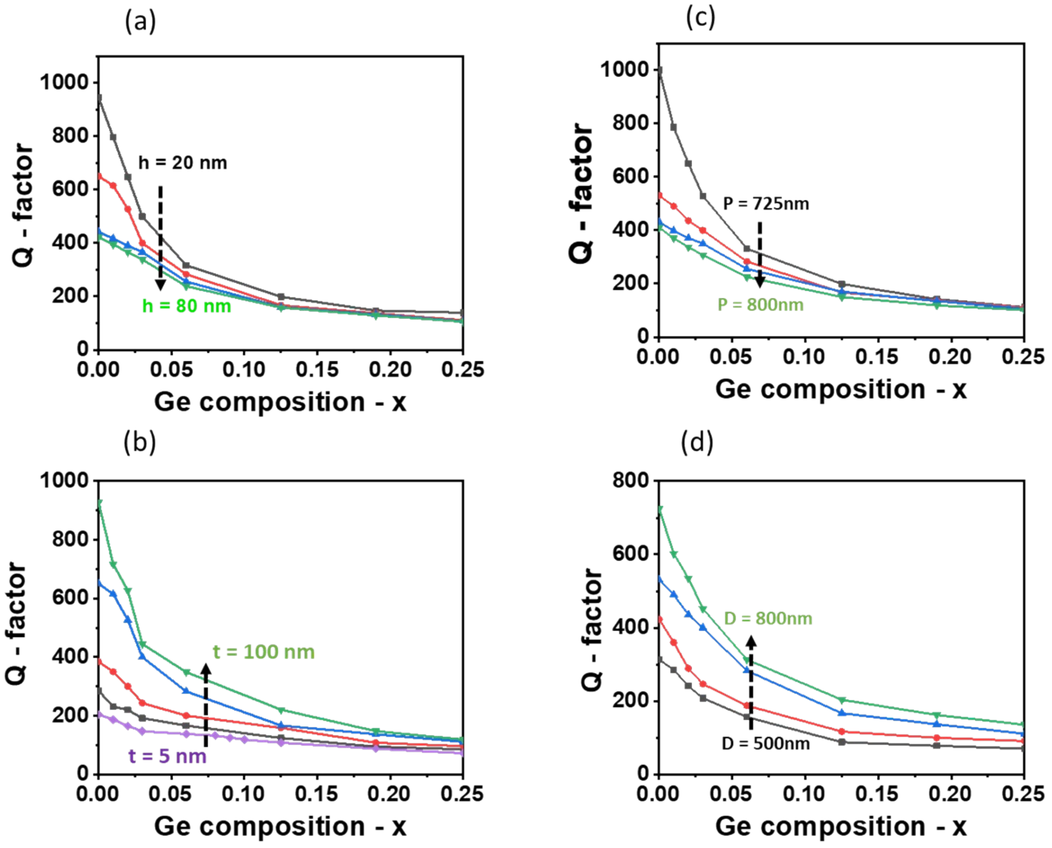

Figure S5. Q-factor of the resonance peak for the following geometries: (a) Disk height (h) varying from 20 nm to 80 nm, with P = 750 nm, D = 700 nm, and t = 75 nm. (b) Thin-film thickness (t), varying from 5 nm to 100 nm, with h = 40 nm, P = 750 nm, and D = 700 nm. (c) Period (P), varying from 725 nm to 800 nm, with h = 40 nm, D = 700 nm, and t = 75 nm. (d) Disk diameter (D), varying from 500 nm to 800 nm, with h = 40 nm, t = 50 nm, and $P = D + 50$ nm.

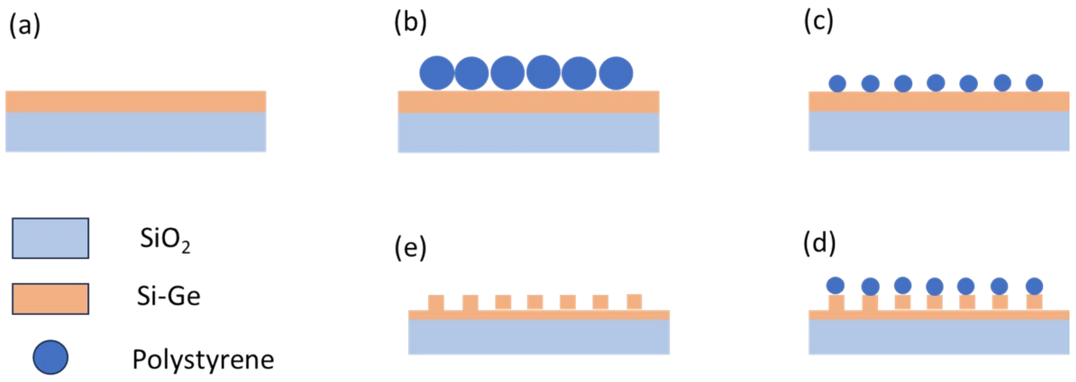

Figure S6. Schematic illustration of the fabrication process of $Si_{1-x}Ge_x$ nanodisk array. A monolayer of polystyrene beads is formed on a sputter-deposited $Si_{1-x}Ge_x$ film. The polystyrene beads are then etched by oxygen plasma until the diameter is reduced to a desired value. The polystyrene beads are used as a photomask for Ar^+-etching of the $Si_{1-x}Ge_x$ film. Finally, polystyrene beads are removed by toluene.

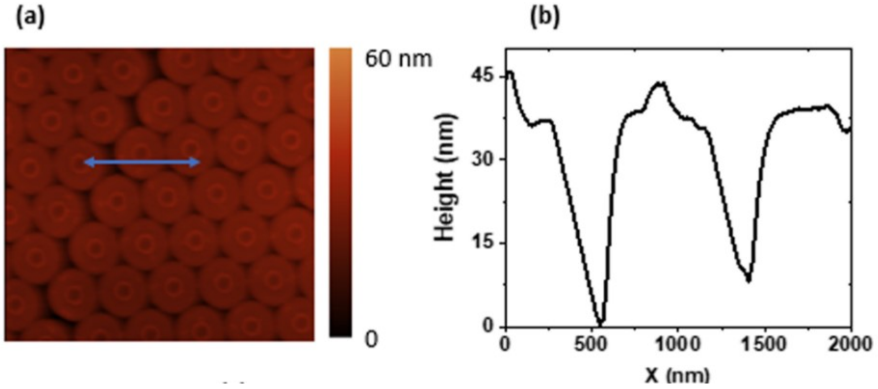

Figure S7. (a) AFM image and (b) the height profile along the arrow in (a) of fabricated $Si_{1-x}Ge_x$ nanodisk array.

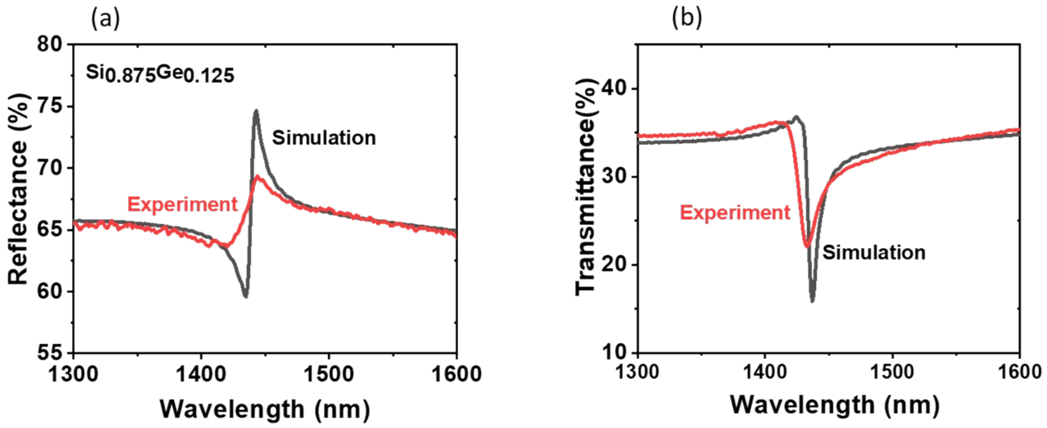

Figure S8. Measured (red) and calculated (black) (a) reflectance and (b) transmittance spectra of $Si_{0.875}Ge_{0.125}$ nanodisk array.

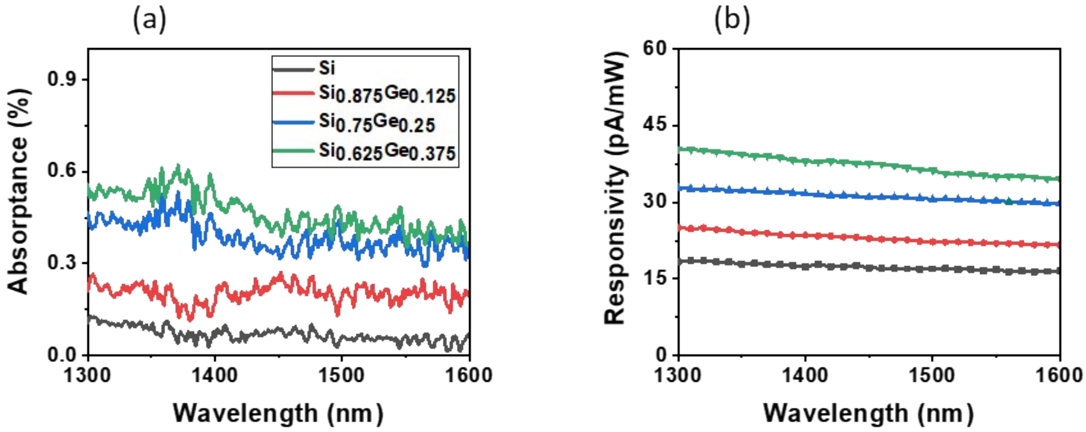

Figure S9. (a) Absorptance and (c) photo responsivity spectra of flat films of $Si_{1-x}Ge_x$ array with x ranging from 0 to 0.375. The films thickness is fixed to 115 nm.

Estimation of Germanium composition in $Si_{1-x}Ge_x$ alloy

Figure S10 shows the Raman scattering spectra of $Si_{1-x}Ge_x$ alloy thin films with different x. Three characteristic peaks corresponding to the Si-Si, Si-Ge, and Ge-Ge vibrational modes are observed. We estimated the composition from the x-dependent shift of the Si–Si peak ($\Delta\omega_{\text{Si-Si}}$) using the following equation [1]:

$$\Delta\omega_{\text{Si-Si}} = -50.6x - 23.74\,x^2$$

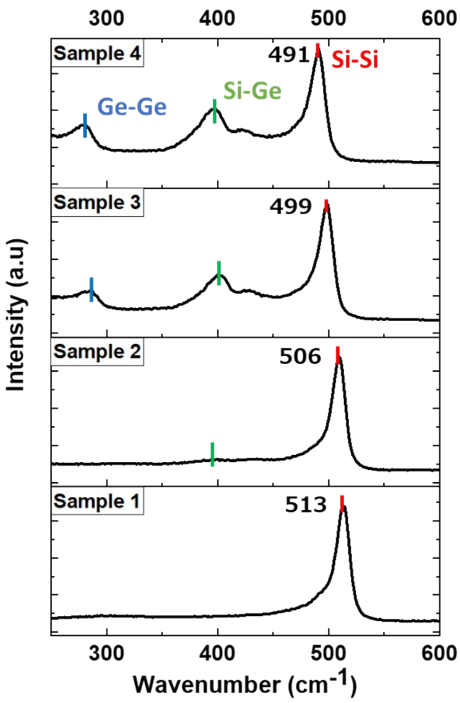

Figure S10. Raman scattering spectra of $Si_{1-x}Ge_x$ alloy thin films with different x. The composition x is estimated from the shift of the Si-Si peak.

Reference

[1] Denis, R. *et al*. Germanium content and strain in Si1−xGex alloys characterized by Raman spectroscopy. *Journal of Crystal Growth* **392,** 66-73 (2014).
